# Path to Facilitate the Prediction of Functional Amino Acid Substitutions in Red Blood Cell Disorders – A Computational Approach

**DOI:** 10.1371/journal.pone.0024607

**Published:** 2011-09-13

**Authors:** Rajith B, George Priya Doss C

**Affiliations:** Medical Biotechnology Division, Center for Nanobiotechnology, School of Biosciences and Technology, Vellore Institute of Technology University, Vellore, Tamil Nadu, India; Institut Jacques Monod, France

## Abstract

**Background:**

A major area of effort in current genomics is to distinguish mutations that are functionally neutral from those that contribute to disease. Single Nucleotide Polymorphisms (SNPs) are amino acid substitutions that currently account for approximately half of the known gene lesions responsible for human inherited diseases. As a result, the prediction of non-synonymous SNPs (nsSNPs) that affect protein functions and relate to disease is an important task.

**Principal Findings:**

In this study, we performed a comprehensive analysis of deleterious SNPs at both functional and structural level in the respective genes associated with red blood cell metabolism disorders using bioinformatics tools. We analyzed the variants in Glucose-6-phosphate dehydrogenase (*G6PD*) and isoforms of Pyruvate Kinase (*PKLR* & *PKM2*) genes responsible for major red blood cell disorders. Deleterious nsSNPs were categorized based on empirical rule and support vector machine based methods to predict the impact on protein functions. Furthermore, we modeled mutant proteins and compared them with the native protein for evaluation of protein structure stability.

**Significance:**

We argue here that bioinformatics tools can play an important role in addressing the complexity of the underlying genetic basis of Red Blood Cell disorders. Based on our investigation, we report here the potential candidate SNPs, for future studies in human Red Blood Cell disorders. Current study also demonstrates the presence of other deleterious mutations and also endorses with *in vivo* experimental studies. Our approach will present the application of computational tools in understanding functional variation from the perspective of structure, expression, evolution and phenotype.

## Introduction

With rapid advances in high-throughput genotyping and next generation sequencing technologies, a vast amount of genetic variation has been discovered and deposited in databases, with much more still to come [1]. One of the major challenges in the analysis of human genetic variation is to distinguish functional from non-functional variants. The simplest form of genetic variation is the substitution of a single nucleotide coined as “Single Nucleotide Polymorphism” (SNPs). SNPs occur at a frequency of approximately to every 100 to 300 base pairs throughout the genome [2]. SNPs that alter the encoded amino acids and might be subjected to natural selection are called non-synonymous SNPs (nsSNPs) and on the other hand, synonymous SNPs do not alter encoded amino acids and are not subjected to natural selection [3]. There is a need to effectively and efficiently identify functionally important nsSNPs which may be deleterious or disease causing and to identify their molecular effects. The prediction of phenotype of nsSNPs by computational analysis may provide a good way to explore the function of nsSNPs and its relationship with susceptibility to disease. For this purpose, a number of bioinformatics tools, based on recent findings from evolutionary biology (amino acid sequence), protein structure research and computational biology may provide useful information in assessing the functional importance of SNPs [4]–[14]. Currently, most molecular studies are focusing on SNPs located in coding and regulatory regions, yet many of these studies have been unable to detect significant associations between SNPs and disease susceptibility. To develop a coherent approach for prioritizing SNP selection for genotyping in molecular studies, we applied an evolutionary perspective to SNP screening. Our hypothesis was that, amino acids conserved across species are more likely to be functionally significant. Therefore, SNPs that change these amino acids might be more likely to be associated with disease susceptibility [15]. It is becoming clear that application of the molecular evolutionary approach may be a powerful tool for prioritizing SNPs to be genotyped in future molecular epidemiological studies [16]–[18]. Therefore, our analysis will provide useful information in selecting SNPs that are likely to have potential functional impact and ultimately contribute to an individual's disease susceptibility.

In recent years, there has been considerable interest in the analysis of Glucose-6-phosphate dehydrogenase (*G6PD*) and Pyruvate Kinase (*PK*) genes for understanding the genetics of Red Blood Cell (RBC) disorders [19]–[21]. Effect of kinetic parameters on over all cellular functions of *G6PD* and PK genes due to change in single nucleotide polymorphism related to human RBC metabolism disorders have already been done [22], [23]. Deficiency in *G6PD* and *PK* genes represents one of the most genetically heterogeneous disorders which lead to chronic anemia with variable severity. *G6PD* deficiency is a sex-linked trait with the gene located on the X-chromosome (band Xq28) about one million base pairs from the telomere end and spans 18 kb. It consists of 13 exons and encodes a mature protein of 530 amino acids [24]. *PK* deficiency is an erythrocyte enzymopathy involving the Embden-Meyerhof pathway of anaerobic glycolysis. PK exists as four isoenzymes namely M1, M2, L and R [25]. PK (L/R) is located on gene locus 1q21 composed of 2 exons spanning 9.5 kb [26] mainly found in liver, normoblasts, reticulocytes, and erythrocytes. PK (M1/M2) is located on gene locus 15q22 composed of 12 exons spanning 32 kb [27] mainly found in striated muscle, brain, fetus, leukocytes, platelets, lungs, spleen, kidneys, adipose tissue etc. *In vivo* and *in vitro* studies on the function of nsSNPs have found that genetic mutations in *G6PD* and *PK* genes are responsible for RBC metabolism disorders [28]–[38]. Validating the known phenotype information gives us a chance to inspect the prediction accuracy. This provides a great opportunity to validate these bioinformatics tools by correlating predicted SNP functional scores to findings from case-control studies [39], [40].

Over the past few years, quite a lot of studies have attempted to identify deleterious nsSNPs within protein-coding sequences, based on sequence information and structural attributes. These methods predict deleterious nsSNPs based on physicochemical properties [41], protein structure [[42]]–[[44], and cross species conservation [18], [42], [43]. The structure of a protein can change in various ways due to the biochemical differences of the amino acid variant (acidic, basic, or hydrophobic), and by the location of the variant in the protein sequence (by affecting tertiary or quaternary structure or the active site where substrate binds). In this light, we employed two diverse approaches in computational analysis of deleterious nsSNPs namely Empirical rule based method and Support Vector Method (SVM). These approaches use alternative classification methods to decide which of the nsSNPs may have deleterious or neutral phenotypes. Empirical rule based method determines parameters manually based on the knowledge of an expert. It is based on the description of SNP in terms of a set of attributes, positional residue variation in sequence alignments, and 3D (three dimensional) structure of the protein and also based on knowledge of the functional site of the proteins. Whereas in SVM approaches, a set of trained data and trained attributes are required to forecast precisely the effects of amino acid substitutions on various protein properties such as protein stability, protein secondary structures, solvent accessibility of residues, residue-residue interactions and protein 3D structures [45]. Hence, we proposed a combinatorial approach using the Empirical rule based and SVM based method to increase the performance of the prediction programs. Knowledge of the 3D structure of a gene product is of major assistance in understanding the function within the cell and its role in causing disease. Proteins with mutations do not always have 3D structures that are analyzed and deposited in Protein data bank (PDB). Therefore, it is necessary to construct 3D models by locating the mutation in 3D structures. This is a simple way of detecting what kind of adverse effects that a mutation can have on a protein. To identify and characterize deleterious mutations in this study, (i) we first surveyed previous publications which genotyped SNPs in case-control studies of *G6PD* and PK deficiency [28]–[38], (ii) analysis of the deleterious nsSNPs was done using Sorting intolerant from tolerant (SIFT) [5], Polymorphism Phenotyping version 1 (PolyPhen) [14], Protein analysis through evolutionary relationship (PANTHER) [7] and I-Mutant 2.0 [46], (iii) the functional SNPs in the untranslated region were analyzed using UTRScan [47] and Functional Analysis and Selection Tool for Single Nucleotide Polymorphism (FASTSNP) [12] (iv) based on SIFT, PolyPhen, PANTHER and I- Mutant 2.0 scores, we identified the possible mutation, proposed a model structure for the mutant proteins and compared this with the native protein in the 3D modeled structure of the *G6PD* and *PK* gene (v) evaluation of modeled structure based on Root mean square deviation (RMSD) and total energy value (vi) identification of stabilizing residues (SRs) in the native and mutant proteins by SRide [48]. Our results, from this study suggest that multiple computational tools should be used when trying to identify deleterious mutations in humans. The goal of the analysis is to predict a deleterious nsSNPs in which there is no availability of 3D structure which are likely to affect the structure or function of the gene and thus to identify which of these SNPs may have possible a role in RBC disorders.

## Results

### Selection of SNPs for analysis

We have selected SNPs from non-synonymous coding region (nsSNPs), coding synonymous region (sSNPs), UTR (5′ and 3′) and intronic region SNPs for our analysis. Out of 539 SNPs, coding region contains 37 (6.9%) non-synonymous SNPs (nsSNPs) and 31 (5.8%) coding synonymous SNPs. Non-coding region contains 414 SNPs (76%) in intronic region and 57 SNPs (10.5%) in mRNA UTR region. It can be seen from the above results that vast majority of SNPs occur in the intronic region. The coding non-synonymous regions and SNPs in regulatory regions were selected for our investigation. The functional impact of nsSNPs can be assessed by evaluating the importance of the amino acids they affect.

### Analysis of deleterious nsSNPs using SIFT

SIFT predicts whether an amino acid substitution affects the protein function based on sequence homology and the physical properties of amino acid**.** Protein sequence with mutational position and amino acid residue variants were submitted as input in SIFT server and the results are outlined in Table S1. A total of 46% nsSNPs were predicted as intolerant by SIFT score of 0.00, 24% of the variants had score ranging from 0.01–0.05 and 30% of the variants exhibited score ranging from 0.06–0.10 respectively. Thus 70% of nsSNPs were predicted to be intolerant, that could bring about changes in protein function. A lower score 0.00–0.05 indicates that the nsSNPs is more damaging to protein function. Such scores enable the quantitative comparison and ranking of SNPs in the order of their biological significance, and are useful for researchers to decide which SNPs of a gene they should first look at.

### Analysis of deleterious nsSNPs using PolyPhen

All protein sequences submitted to SIFT were also submitted to PolyPhen. Unlike SIFT, it does not solely depend on sequence homology alone to make SNP functional predictions as its modeling of the amino acid substitutions is also based on structural information. The PolyPhen output comprises a score that ranges from 0 to a positive number, with zero indicating a neutral effect of amino acid substitutions on protein function. Conversely, a large positive number indicates that the substitution is detrimental to protein function. According to PolyPhen score, 33% of the nsSNPs were found to be “Possibly damaging” to protein structure and function; 12% of the nsSNPs exhibited PolyPhen scores of 1.99–1.50 indicating that the variants were “Possibly damaging” to protein function and the remaining 55% were characterized as benign (Table S1). Such scoring is once again useful as ranking of SNPs according to their significance can be carried out to enable quantitative assessment of the severity of the effect on protein function.

### Identification of functional nsSNPs using I-Mutant 2.0

The protein stability change due to single point mutation was predicted using support vector machine-based tool I-Mutant 2.0. All the nsSNPs submitted to SIFT and PolyPhen were also submitted as input to the I-Mutant 2.0. According to I-Mutant 2.0, more negative the free energy value (DDG value); less stable the given point mutation is likely to be. Out of 37 variants, 4 variants (L323P, I48T, V68M and Q310P) which showed DDG value of −2.07, −2.05,−3.32 and −2.20 respectively, were considered to be least stable and deleterious. The other 11 variants R285H, R459P, R463H, D350H, D181V, D113N, Q11H, N126D, S437V, G200C and R339P showed DDG value between −1.00 to −2.00 and the remaining 15 variants showed DDG value less than 0.00 (Table S1).

### Prediction of deleterious nsSNPs using PANTHER

All the nsSNPs with maximum significant score using SIFT, PolyPhen and I-Mutant 2.0 were further analyzed using PANTHER for validating its impact on protein function upon single point mutation. PANTHER is able to classify proteins by function, thus adding another layer of complexity to refine SNP prediction. This tool is able to generate a variety of outputs, the most useful being the probability that a particular variant is deleterious. Out of 37 nsSNPs taken for our analysis, 8 nsSNPs with subPSEC score less than -5 were designated as highly deleterious, out of which the amino acid substitution position at A44G, R163C and Q310P showed a good correlation with SIFT, PolyPhen and I-Mutant 2.0 scores respectively. Hence the following SNPs with ID rs78478128, rs74315362 and rs11558370 having subPSEC scores −5.29666, −8.05846 and −7.51475, were selected for further modeling analysis. The remaining 18 variants having subPSEC score less than -3 were found to be deleterious and eleven nsSNPS with subPSEC score greater than -3 were predicted to be intolerant using PANTHER (Table S1). The nsSNPs which were predicted to be deleterious in causing an effect in the structure and function of the protein by SIFT, PolyPhen, I-Mutant 2.0 and PANTHER correlated well experimental studies as shown in Table S1 [[28]]–[[38].

### Combination of the Prediction Programs for deleterious nsSNPs

**Figure 1:**
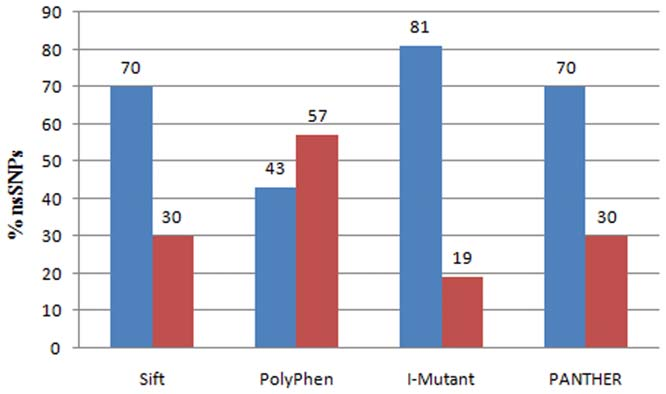
Distribution of predicted nsSNPs in Glucose-6-phosphate dehydrogenase and Pyruvate kinase genes. Bar diagram displays the percentage (%) of deleterious and benign nsSNPS by SIFT, PolyPhen, I-Mutant-2.0 and PANTHER. Blue rectangle bar indicates percentage of nsSNPs found to be deleterious by SIFT and PANTHER, damaging (Possibly/Probably) by PolyPhen, and decrease stability by I-Mutant-2.0. Red rectangle indicates percentage of nsSNPs tolerated by SIFT and PANTHER, benign by PolyPhen, and increase stability by I-Mutant-2.0.

Prediction of deleterious nsSNPs can be made more accurate by combining different computational methods [12], [47]. Thus, we combined Empirical and SVM based prediction programs, and found that this could significantly increase prediction performance in deleterious nsSNPs analysis. We used the SIFT, PolyPhen, I-Mutant 2.0 and PANTHER programs to predict the influence of nsSNPs on protein function and structure. Figure 1 shows the distribution of predicted scores of SIFT, PolyPhen, I-Mutant 2.0 and PANTHER. Since a lower SIFT or PANTHER or I-Mutant 2.0 score indicate that the nsSNP of interest would be more deleterious, whereas a higher PolyPhen score indicate that the nsSNPs of interest would be more deleterious. Out of 37 nsSNPs, 26 nsSNPs were predicted to be deleterious by SIFT, PolyPhen predicted 16 nsSNPs as damaging, 26 nsSNPs as deleterious by PANTHER and 30 nsSNPs as less stable by I-Mutant 2.0. Out of all predictions, 70%, 43%, 70%, and 80% were specific to SIFT, PolyPhen, PANTHER and the I-Mutant 2.0, respectively, and 9 nsSNPs (24%) were predicted to be functionally significant by all four methods. We found that I Mutant 2.0 was able to predict 80% deleterious nsSNPs, slightly higher than SIFT (70%) and PANTHER (70%). However, PolyPhen predicted only 43% of deleterious nsSNPs which uses a normalized cross-species conservation score and combines this with a variety of protein structural features when available. Most of these differences are likely the result of each method requiring a sufficient number and diversity of aligned sequences in order to make a prediction, each method using a different set of sequences and alignments. We have shown that our data suggests that individual tools correlate modestly with observed results, and that combining information from a variety of tools may significantly increase the predictive power for determining the functional impact of a given nsSNP.

### Characterization of functional regulatory elements in 5′ and 3′ untranslated region by UTRscan

In this method, the effect of UTRs on phenotypic variation has been elucidated with the help of patterns of functional sequences deposited in the UTRSite and UTRdb. In this method, the integration of genomic data with UTRdb transforms it into an efficient annotation technique as well as a powerful retrieval resource for the UTR subsets. The UTRscan server however did provide some useful indicators with respect to the functional effects of 3′ and 5′ UTR SNPs of *G6PD* and *PK* genes. All the 57 UTR SNPs (3′ and 5′) were analyzed using UTRscan. After comparing the functional elements for each UTR SNPs, we found 11 SNPs in the 3′ UTR and 12 SNPs in 5′ UTR have different functional pattern(s) for each sequence, and were predicted to have functional significance. Totally, 23 SNPs in both 3′ and 5′ regions were found to be functionally significant as shown in Table S2. Among these SNPs, 9 of them were related to the functional pattern change of Internal Ribosome Entry Site (IRES) [49], 10 of them were related to the functional pattern change of Musashi binding [50], [51], 4 of them related to functional pattern change of K-Box, 3 of them related to functional pattern change of Terminal oligopyramidine track [52] and one of them were related to functional pattern change of SXL (Sex-lethal) binding site [53], Selenocysteine insertion sequence type 1 [SECIS] [54], UNR binding site [55], Polyadenylation signal respectively.

### Prediction of functional nsSNPs by FASTSNP

FASTSNP tool help in classifying and prioritizing phenotypic risks and deleterious effects of SNPs based up on their influence over determining protein structure, pre-mRNA\splicing, deviation in transcriptional levels of the sequence, alterations in the premature translation termination, deviations in the sites at promoter region for transcription factor binding, etc. However, FASTSNP could not predict the functional importance of the SNPs in the 5′ and 3′ regions. 25 nsSNPs were found to be functionally significant in coding region. Out of which 10 SNPs were predicted to affect the splicing site with a risk ranking of 3–4, 14 SNPs were predicted to affect splicing regulation with a risk ranking of 2–3 and 1 coding nonsense SNP (rs11558352) was detected with a very high (5–5) level of risk, as it can truncate and even inactivate the PKM2 protein, causing diseases (Table S3).

### Modeling of deleterious nsSNPs

Single amino acid mutations can significantly change the stability of a protein structure. So, the knowledge of a protein's 3D structure is essential for a full understanding of its functionality. Mapping the deleterious nsSNPs into protein structure information was obtained from dbSNP and SAAPdb [56]. Mutation analysis was performed based on the results obtained from highest SIFT, PolyPhen, I-Mutant 2.0 and PANTHER scores. The mutations at their corresponding positions were performed by SWISS-PDB viewer independently to achieve modeled structures. Then, energy minimizations were performed by NOMAD-Ref server for the native type protein and mutant type structures.

### Glucose 6 Phosphate Dehydrogenase (*G6PD*)

**Figure 2:**
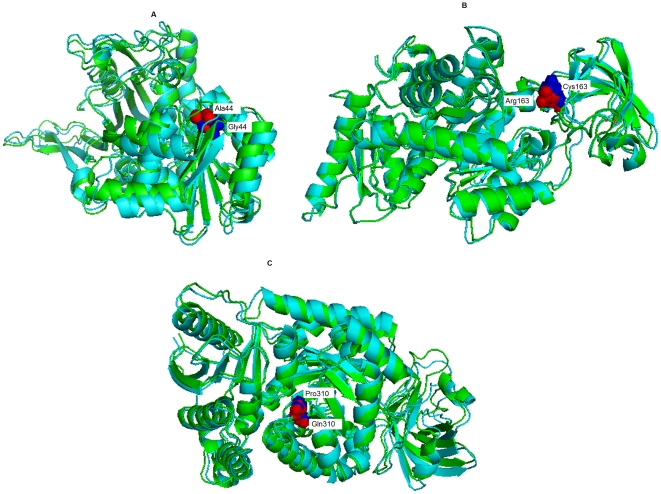
Superimposition of native and mutant modeled structures (cartoon shape) of *G6PD*, *PKLR* and *PKM2* genes. (A). Superimposed structure of native amino acid Alanine in sphere shape (red color) with mutant amino acid Glycine (blue color) at position 44 in PDB ID 2BHL of *G6PD* gene with RMSD 1.88 Å. (B). Superimposed structure of native amino acid Argenine in sphere shape (red color) with mutant amino acid Cysteine (blue color) at position 163 in PDB ID 2VGB of *PKLR* gene with RMSD 2.82 Å. (C). Superimposed structure of native amino acid Glutamine sphere shape (red color) with mutant amino acid Proline (blue color) at position 310 in PDB ID 1T5A of *PKM2 gene* with RMSD 2.8 Å.

In *G6PD* gene, mutation occurred for native protein in ‘A’ chain of PDB ID [2BHL] at position A44G with SNP ID rs78478128 and at R459P with SNP ID rs72554665. It can be seen that the total energy value of native type (−25480.939 Kcal/mol) and mutant modeled structure A44G and R459P were found to be −25299.660 Kcal/mol and **−**25296**.**779 Kcal/mol respectively. The RMSD value between the native and the mutant A44G, R459P were 1.88 Å and 1.74 Å. The superimposed structures of the native protein 2BHL (chain A) with the mutant type proteins A44G of *G6PD* gene is shown in (Figure 2A).

### Pyruvate Kinase isoforms (*PKLR* & PKM2)

In *PKLR* gene, mutation occurred for native protein in ‘A’ chain of PDB ID [2VGB] at position R163C with SNP ID rs118204083, R486W with SNP ID rs116100695 and T384M with SNP ID rs74315362. It can be seen that the total energy value of native type and mutant modeled structure R163C, R486W and T384M were found to be −24360.674 Kcal/mol and −24081.020 Kcal/mol, **−**24090.248 Kcal/mol, −24084.902 Kcal/mol respectively. The RMSD values between the native and the mutant type R163C, R486W and T384M were found to be 2.82 Å, 2.74 Å and 2.52 Å. Similarly in *PKM2* gene, mutation occurred at ‘A’ chain of PDB ID [1T5A] at positions C31F with ID rs11558375, G200C with ID rs11558354 and Q310P with ID rs11558370. The total energy value were found to be −24868.992 Kcal/mol for native protein and −24623.072 Kcal/mol, −24654.807 Kcal/mol, −24608**.**215 Kcal/mol for mutant type C31F, G200C and Q310P respectively Their respective RMSD value were found to be 2.45 Å, 2.4 Å, 2.8 Å. Higher the RMSD value more will be the deviation between native and mutant type structures and which in turn changes their functional activity. The superimposed structures of the native protein 2VGB with the mutant type protein R163C, of *PKLR* gene is shown in (Figure 2B) and the superimposed structure of native protein 1T5A and mutant type model Q310P of *PKM2* gene is shown in (Figure 2C). These figures were drawn using PyMOL54 release 0.99 [57]. Green color cartoon model by PyMOL54 release 0.99 represents the native structure while blue color cartoon model represents mutant modeled structure respectively.

### Computation of stabilizing residues in native and mutant modeled structures of *G6PD* and *PK* genes

We used the SRide server for identifying the SRs in native type structure and mutant modeled proteins in *G6PD*, *PKLR*, and *PKM2* genes (Table 1). From this analysis four SRs were found to be common in both native structure (2BHL) and mutant model R459P of *G6PD* gene. In mutant model R459P, three stabilizing residues were found namely Ala338, Gln372, and Gln308, which revealed that these stabilizing residues could be the major factor in destabilizing the 2BHL structure. In *PKLR* gene all the four SRs, Ile90, Cys401, Ala495, Ile553 respectively were identical in native protein (2VGB) and mutant models R163C, R486W and T384M. Four SRs (Gly122, Cys358, Ala452 and Ile510**)** were identified to be identical in native type protein (IT5A) and mutant model G200C and Q310P of *PKM2* gene. In mutated model (C31F), one additional residue Cys49 was found which may play key factor in destabilizing the structure.

**Table 1:** Computing stabilizing residues (SRs) in native and mutant proteins of *G6PD, PKLR*, and *PKM2* genes using SRide.

Gene	Stabilizing residues in native protein	Stabilizing residues in mutant proteins		
*G6PD* (2BHL)	Ala141,Gly306, Gln307, Tyr308	2BHL (A44G)	2BHL (R459P)	
		Ala141, Gly306, Gln307, Tyr308	Gly306, Tyr308, Ala338, Gln372, Gln308	
*PKLR* (2VGB)	Ile90, Cys401, Ala495, Ile553	2VGB (R163C)	2VGB (R486W)	2VGB (T384M)
		Ile90, Cys401, Ala495, Ile553	Ile90, Cys401, Ala495, Ile553	Ile90, Cys401, Ala495, Ile553
*PKM2* (IT5A)	Gly122, Cys358, Ala452, Ile510	IT5A (C31F)	IT5A (G200C)	IT5A (Q310P)
		Cys49, Gly122, Cys358, Ala452, Ile510	Gly122, Cys358, Ala452, Ile510	Gly122, Cys358, Ala452, Ile510

Residues marked in bold were found to be common in both native and mutant type structures.

## Discussion

For reliable information about the protein, a sequence variation is essential to gain insights into disease genotype–phenotype correlations. How is it possible to discriminate between amino acid substitution that are deleterious for the stability or for the function of the protein, leading to a disorder, and neutral variations that do not modify the phenotype? An increasing number of computational approaches to *in silico* analysis of substitutions available on the World Wide Web have been proposed to answer this question [[4]]–[[14]. Over the past five years, computational approaches to *in silico* analysis of amino acid substitutions have improved considerably. In this aspect, we have brought *in silico* model with two diverse approaches (Empirical and SVM) in forefront to experimental biologists as an alternative method in determining the functional SNPs in RBC disorders. Both these diverse methods use sequence information, structural information or both. Sequence (SIFT, PANTHER) and structure based methods (PolyPhen, I-Mutant 2.0) are the most common approaches used in SNP prediction tools. Sequence-based prediction methods have more advantage over the structure-based ones, as they include all types of effect at the protein level, and can be applied to any human protein with known relatives. However, sequence-based predictions (based on homology and evolutionary conservation) are unable to reveal the underlying mechanisms of how SNPs result in changed protein phenotypes. On the other hand, structure-based approach is not feasible to implement for the proteins with unknown 3D structures. Thus, structure based approach has limited applicability. Tools that integrate both sequence and structure information have the added advantage of being able to assess the reliability of the generated prediction results by cross-referencing the results from both approaches. Tools that combine these approaches (PolyPhen and I-Mutant 2.0) use different algorithms and methodologies for prediction, thereby having a wider cover-age of the different aspects of SNP analysis. Both the methods have disadvantages and advantages in predictng the effects of SNPs on protein stability. The user must decide which tool is most suited to the specific objectives of their analysis to gain the optimum knowledge. Although the predictive power of protein structural information has been established, a comparison between structure-based and sequence based methods is still needed.

Taking into this account, we focused our attention by comparing the prediction functionality of curated amino acid substitutions in *G6PD, PKLR* and *PKM2* associated with RBC disorder genes using existing computational methods SIFT, PolyPhen, I-Mutant-2.0 and PANTHER. In addition, we used FASTSNP and UTRscan for each gene to evaluate role of SNPs in regulatory regions. From the results obtained, SIFT predicted 70% of nsSNPs to be deleterious and PolyPhen predicted 43% of nsSNPs to be damaging. A combination of SIFT and PolyPhen tools were able to predict only 40% of deleterious nsSNPs in *G6PD* and *PK* genes. Karchin et al. [9] argued that when the outputs from the algorithms SIFT and PolyPhen differ, it is more likely due to using different protein sequence alignments compared to the differences in scores used to classify the variants. Several groups have tried to evaluate the ability of SIFT to distinguish between neutral and deleterious substitutions [42], [43], [61]. The performance of SIFT was also analyzed in healthy individuals by Cargill et al. [58]. In another set of studies, Palmer et al. tried to validate the SIFT in *MSHR* gene, and found that predicted tolerated substitutions L60V and R163Q by SIFT were in concordance with the experimental results [59]. To date, data on the validity of these algorithms has come from benchmarking studies based on the analysis of “known” deleterious substitutions annotated in databases, such as SwissProt, [40], [43], [60], [61]. Experimental studies of individual proteins have also confirmed the accuracy of SIFT [62]–[64]. Our group also tried to evaluate the accuracy of SIFT and PolyPhen based predictions on *CFTR, PAH, HBB*, *TP53, HNPCC* genes [65]–[69]. We observed good concordance between SIFT and PolyPhen methods. In this analysis, we tried to evaluate the Hidden Markov Model (HMM) based PANTHER and SVM based I-Mutant 2.0 as additional tools in identifying deleterious substitutions. We used PANTHER that uses scoring matrices which were similar to the SIFT approach. The primary mission of the PANTHER database is to organize genes into families and subfamilies and to classify them according to inferred function. Much of the organization achieved by this database relies on making protein multiple sequence alignments (PMSAs) across a large number of gene subfamilies and families. The proportion of deleterious nsSNPs predicted by PANTHER (70%) was similar to SIFT (70%) in *G6PD* and *PK* genes. When we analyzed the deleterious nsSNPs predicted by the combination of SIFT vs PolyPhen, SIFT vs PANTHER and PolyPhen vs PANTHER combination, the prediction of SIFT vs PANTHER was highest among all (40%, 60% and 30%). To improve the strength of our analysis, we lastly compared I-Mutant to SIFT, PolyPhen and PANTHER. I-Mutant is a machine learning-based method that takes as input, protein sequence, protein structure, and protein function information. I-Mutant 2.0 predicted 80% of nsSNPs to be deleterious. The proportion of deleterious nsSNPs predicted by I-Mutant vs PANTHER (60%) was higher than the combinations of SIFT vs I-Mutant and PolyPhen vs I-Mutant respectively (60% and 30%). Significant concordance was observed between the functional consequences of nsSNPs predicted by various combinations of the tools. The prediction accuracy of deleterious nsSNPs by I-Mutant 2.0 is much higher than other three tools used in our analysis. Recent analysis by capriotti and Atman [70] also stated that the prediction accuracy of SVM based method I-Mutant 2.0 was higher with respect to SIFT and PolyPhen. By comparing the scores of all the four methods used in this analysis, 9 nsSNPs (24%) with IDs rs17853396, rs2959910, rs11558354, rs11558370, rs11558375, rs118204083, rs117089358, rs72554665 and rs78478128 were predicted to be functionally significant.

The 5′ and 3′ untranslated regions of eukaryotic mRNAs (UTRs) play crucial roles in the post-transcriptional regulation of gene expression [47]. The functional prediction of SNPs in untranslated region for the *G6PD*, *PKLR*, and *PKM2* genes have not been estimated computationally until now, although they have been the focus for experimental researchers. Therefore in this work, we used FASTSNP and UTRscan for this analysis. By FASTSNP 25 nsSNPs were found to be functionally significant in the coding region. Further, we extended our analysis by comparing FASTSNP with SIFT, PolyPhen, I-Mutant 2.0 and PANTHER. Out of 25 nsSNPs predicted by FASTSNP, 20 nsSNPs were found to be deleterious by SIFT/PolyPhen/PANTHER/I-Mutant 2.0 highlighted as bold in Table S3. The FASTSNP server could not predict the functional impact of SNPs in the 5′ and 3′ region. After comparing the functional elements for each SNPs in the untranslated regions using UTRscan, we found 11 SNPs in the 3′ and 12 SNPs in 5′ untranslated region were predicted to have functional significance due to the presence of different functional pattern(s) for each sequence. The functional pattern change includes IRES, Musashi binding, SXL binding site, SECIS, UNR binding site, Polyadenylation signal respectively. IRES are bound by internal mRNA ribosome. The IRES is believed to be involved in internal mRNA ribosome binding, which allows for translation to occur during periods of the cell cycle when the conventional mechanism of translation is ineffective. It is an alternative mechanism of translation initiation compared to the conventional 5′-cap dependent ribosome scanning mechanism [49]. Musashi binding protein plays an important role in regulating the expression of target mRNAs at the translation level. It may also play a role in the proliferation and maintenance of stem cells in the central nervous system [50], [51]. SXL binding site plays an important role in regulating splicing of specific target genes by directly interacting with their pre-mRNAs and also for better understanding of translation repression [53]. The SECIS element is a specific 60 bp stem-loop structure located in 3′UTRs of mRNAs, and required for decoding UGA selenocysteine instead of termination of translation [54]. Terminal oligopyramidine tract encode for ribosomal proteins and elongation factors 1alpha and 2, and are candidates for growth-dependent translational control [52]. The SNPs with IDs rs5030868, rs1050828 and rs1050829 predicted by UTRscan were well supported by experimentally methods [71], [72].

Protein structural analysis were carried out based on the screened results obtained from SIFT, PolyPhen, I-Mutant-2.0 and PANTHER. Protein 3D structural information is an important feature for predicting the effects of deleterious nsSNPs. Analysis of the protein structure provides information about the environment of the mutation. Proteins with mutations do not always have 3D structures that are analyzed and deposited in PDB. Therefore, it is necessary to construct 3D models using molecular modeling protocols. This is a simple way of detecting what kind of adverse effects that a mutation can have on a protein. Single amino acid substitution were examined using Swiss-PDB viewer with RMSD value computed from mutant and native structure used in predicting the deviation. Computing the energy gives the information about the protein structure stability. We compared RMSD value and Total energy values (Kcal/mol) of Native structure and mutated modeled structure for the three genes. Mutant structures of *G6PD* with PDB ID 2BHL at position A44G, mutant structures of *PKLR* with PDB ID 2VGB at position R163C and mutant structures of PKM2 with PDB ID 1T5A at position Q310P showed an increases in total energy level (less favorable change) and increase in RMSD value deviation in comparison with native structure. Divergence in mutant structure with native structure is due to mutation, deletions, and insertions [73] and the deviation between the two structures is evaluated by their RMSD values which could affect stability and functional activity [74]. These results were in very well concordance with SIFT, PolyPhen, I-Mutant-2.0 and PANHTER scores. Computational analysis of stabilizing residues which plays an important role in stabilization of protein was carried out using SRide tool. Interestingly our approach identified three SRs, Alanine at position 338 and Glutamine at position 372 and 308 plays an important role in destabilizing the mutant structure (PDB ID 2BHL) of *G6PD* gene. Similarly amino acid Cysteine plays an important role destabilizing the mutant structure (PDB ID IT5A) of *PKM2* gene. The overall approach of our study was to prioritize the functional nsSNPs, map as many structural mutations as possible, find general patterns to analyze 3D mutations with regard to protein function and evaluate regulatory variants using as many bioinformatics analysis methods as possible. Based on these analyses, we attempt to establish the relationship between the disease-related mutations and structural properties of proteins. The result from this work suggests that combination of SVM based I-Mutant 2.0 increases the accuracy of the prediction of deleterious nsSNPs. The methods which are adopted in this study uses web-based interface that are easy to use for the beginner. The inputs are standard sequence formats or ID numbers as well as the SNP information. The computational approach proposed in this study is based on integrating relevant biomedical information sources to provide a systematic analysis of functional and deleterious nsSNPs associated with RBC disorders. Various computational tools used in this analysis determine the functional effects of SNPs only with respect to a single biological function. Therefore, much time and effort is required from researchers to identify the appropriate tools and interpret the predictions. As there is a vast number of SNPs, it might not be feasible for researchers to carry out wet laboratory experiments on every SNP to determine their biological significance. These methods will benefit researchers to prioritize amino acid substitutions as it would be time consuming, difficult, and expensive to experimentally characterize the impact of each nsSNPs on protein function.

## Analysis

### Data mining

The data on human *G6PD*, *PKLR* and *PKM2* genes were collected from Online Mendelian Inheritance in Man (OMIM) [75] and Entrez Gene on National Center for Biological Information (NCBI) Web site. The SNPs information (Protein accession number (NP), mRNA accession number (NM) and SNP ID) of *G6PD*, *PKLR* and *PKM2* genes were retrieved from the NCBI dbSNP (http://www.ncbi.nlm.nih.gov/snp/), and SWISS-Prot databases (http://expasy.org/) [76] for our computational analysis. The information on the effect of the nsSNP variants and the correlation between the nsSNP and disease was compiled from *in vivo* and *in vitro* experiments according to PubMed (www.ncbi.nlm.nih.gov/PubMed/), OMIM (www.ncbi.nlm.nih.gov/omim/), and UniProtKB/Swiss-Prot databases (ca.expasy.org/sprot/).

### Sequence homology based method

SIFT uses sequence homology to predict whether an amino acid substitution will affect the protein function [5]. SIFT (http://sift.jcvi.org/www/SIFT_dbSNP.html) is a multistep process which starts with selection of sequences that are similar to query protein sequence, make an alignment of these sequences and calculating scores based on amino acids appearing at each position in the alignment. Several versions of this program have been described and differ in the protocol used to select homologous sequences. The most recent version SIFT BLink Beta was utilized in this study. We performed SIFT by submitting query in the form of gene identification number obtained from NCBI. SIFT score provides the tolerance index of a particular amino acid substitution to protein function. The underlying principle of this program is that it generates alignments with a large number of homologous sequences and assigns scores to each residue, ranging from zero to one. SIFT score ≤0.05 indicates the amino acid substitution is intolerant or deleterious, where as score ≥ 0.05 is predicted to be tolerant. The alignment built by SIFT contains homologous sequences with a medium conservation measure of 3.0 where conservation is represented by information content [77] to minimize false positive and false negative error. SIFT also warns that median sequence information above 3.25 represents sequences that are very similar and may lead to false results. We used the default median sequence conservation in the range of 3.0.

### Structural Homology based method

PolyPhen is a software tool which predicts possible impact of amino acid substitutions on the structure and function of human proteins using straight forward physical and evolutionary comparative considerations [14]. The input to PolyPhen is an amino acid sequence or corresponding ID, the position of the amino acid varied, and the amino acid variants. The prediction is based on straightforward empirical rules that are applied to the sequence, phylogenetic and structural information characterizing the substitution. Input for the PolyPhen server (http://genetics.bwh.harvard.edu/pph/) is either a protein sequence or a SWALL database ID or accession number together with sequence position with two amino acid variants. We submitted the query in the form of amino acid sequence in FASTA sequence with mutational positions each with two amino acids variants. PolyPhen searches for 3-D protein structures, multiple alignments of homologous sequences and amino acid contact information in several protein structure databases. Then, it calculates PSIC scores for each of two variants, and computes the difference of the PSIC scores of the two variants. The higher a PSIC score difference, the higher is the functional impact a particular amino acid substitution is likely to have. A PSIC score difference of 1.5 and above is considered to be damaging. The PolyPhen scores can de classified as probably damaging (≥2.00), possibly damaging (1.50–1.99), potentially damaging (1.25–1.49), or benign (0.00–0.99).

### I-Mutant 2.0

We used I-Mutant 2.0 available at www.gpcr.biocomp.unibo.it/cgi/predictors/I-Mutant 2.0.cgi to calculate the stability of mutated proteins. I-Mutant 2.0 is a SVM-based method for the automatic prediction of protein stability changes upon single point mutations. I-Mutant 2.0 can be used as a unique and valuable helper for protein designing, even when the protein structure is not yet known with atomic resolution. There are two alternatives available for the input data in I-Mutant 2.0, predicting the protein stability change upon single point mutation using protein structure or protein sequences [46]. This program was trained and tested on a dataset derived from ProTherm [78], which is the most comprehensive available database of thermodynamic experimental data of free energy changes of protein stability upon mutation under different conditions. The output file shows the predicted free energy change (DDG) which is calculated from the unfolding Gibbs free energy change of the mutated protein minus the unfolding free energy value of the native protein (Kcal/mol). We performed the analysis using protein sequence data available from dbSNP data base. DDG >0 means that the mutated protein has high stability and vice versa.

### PANTHER

PANTHER is a database which contains a collection of protein families and subfamilies that predicts how often does a given amino acid occurs at a given position in a family of evolutionary related protein across different species [7]. It uses HMM-based statistical modeling methods and multiple sequence alignments to perform evolutionary analysis of coding nsSNPs. By calculating the substitution position-specific evolutionary conservation score (subPSEC) based on an alignment of evolutionarily related proteins, PANTHER estimates the likelihood of a particular nsSNP causing a functional impact on the protein PANTHER subPSEC scores vary from 0 (neutral) to about −10 (most likely to be deleterious). Protein sequences having subPSEC value ≤ −3 is said to be deleterious.

### SRide

SRide is a server for identifying the stabilizing residues (SRs) in protein, located at http://sride.enzim.hu/. A residue is said to be stabilizing, if it has high surrounding hydrophobicity (Hp), high long range order (LRO), high conservation score and if it belongs to a stabilizing center (SC) [48]. The algorithm for identifying SRs is now generalized for all proteins of known 3D structure. The input for SRide server is either PDB ID or by uploading the protein structure in PDB format.

### Modeling of mutant protein structures

SNP can significantly change the stability of proteins. So, for understanding the significance of a single nucleotide change in protein function, knowledge about 3D structure of protein is very important. Structure analysis was performed for evaluating the structural stability of native and mutant protein. We used the dbSNP to identify the protein coded by *G6PD* (PDB ID 2BHL) and *PK* genes (PDB ID 2VGB). We also confirmed the mutation positions and the mutation residues from this server. These mutation positions and residues were in complete agreement with the results obtained with SIFT and PolyPhen programs. The mutation analysis was performed using SWISSPDB viewer, and energy minimization for three-dimensional structures was performed using NOMAD-Ref server [79]. This server use Gromacs as default force field for energy minimization based on methods of steepest descent, conjugate gradient and L-BFGS methods [80]. We use conjugate gradient method for optimizing the 3D structures. Computing the energy gives the information about the protein structure stability. Deviation between the two structures was evaluated by their RMSD values.

### FASTSNP

In order to efficiently identify nsSNPs with a high possibility of having a functional effect, FASTSNP tool was applied for the detection of nsSNP influence on cellular and molecular biological function e.g. transcriptional and splicing regulation. The online tool FASTSNP [12] (http://fastsnp.ibms.sinica.edu.tw/pages/input_CandidateGeneSearch.jsp) was used for predicting the functional significance of the nsSNPs, 3′ and 5′ UTR SNPs and also to identify the polymorphism involving intron which may lead to defects in mRNA processing. The FASTSNP follows the decision tree principle with external web service access to TFSearch, which predicts whether a non-coding SNP alters the transcription factor binding site of a gene. The score is given on the basis of levels of risk with a ranking of 0, 1, 2, 3, 4, or 5. This signifies the levels of no, very low, low, medium, high, and very high effect, respectively.

### UTRscan

UTRscan (http://itbtools.ba.itb.cnr.it/utrscan) was used for characterization of SNPs in regulatory untranslated regions [47]. This tool was used to analyze the untranslated regions (5′ UTR and 3′UTR) of eukaryotic mRNA which are involved in many post transcriptional regulatory pathways that control mRNA localization, stability and translational efficiency [69]. The internet resource for UTR analysis are UTRdb, which contains experimentally proven biological activity of functional pattern of UTR sequence from eukaryotic mRNAs and UTRsite, which is a collection of functional sequence patterns located in 5′ or 3′ UTR sequence. Briefly, two or three sequences of each UTR SNP that have a different nucleotide at an SNP position are analyzed by UTRscan, which looks for UTR functional elements by searching through user-submitted sequence data for the patterns defined in the UTRsite and UTR databases. If different sequences for each UTR SNP are found to have different functional patterns, this UTR SNP is predicted to have functional significance.

## Supporting Information

Table S1 Summary of nsSNPs that were analyzed by four computational methods SIFT (Tolerated/Deleterious), PolyPhen (Benign/Damaging), I-Mutant (Increase stability/Decrease stability and PANTHER (Tolerated/Deleterious).
(DOC)

Table S2 Functional significance of SNPs found in untranslated region of *G6PD* and isoforms of *PK* genes by UTRscan.
(DOC)

Table S3 List of nsSNPs and UTR SNPs found to be functionally significant by FASTSNP.
(DOC)
