# Supplementary material for: Path to Facilitate the Prediction of Functional Amino Acid Substitutions in Red Blood Cell Disorders – A Computational Approach
Source: PLoS One. 2011 Sep 13;6(9):e24607. doi: 10.1371/journal.pone.0024607 (PMC3172254; doi:10.1371/journal.pone.0024607)
Supplement: Table S1 — Summary of nsSNPs that were analyzed by four computational methods SIFT (Tolerated/Deleterious), PolyPhen (Benign/Damaging), I-Mutant (Increase stability/Decrease stability and PANTHER (Tolerated/Deleterious). (DOC) [file pone.0024607.s001.doc]

**Table S1. Summary of nsSNPs that were analyzed by four computational methods SIFT (Tolerated /Deleterious), PolyPhen (Benign/Damaging), I-Mutant (Increase stability/Decrease stability and PANTHER (Tolerated/Deleterious).**

**AA-Amino Acid; NA-Not available; SIFT Prediction score: Deleterious (≤ 0.05); Tolerated (≥.0.05); PolyPhen Prediction Score: Damaging (≤1.5); Benign (≥1.5); I-Mutant 2.0 Prediction score: Decrease stability**

|  |  |  |  | **SIFT** | | **PolyPhen** | | **I-Mutant 2.0** | | **PANTHER** | |  |
| --- | --- | --- | --- | --- | --- | --- | --- | --- | --- | --- | --- | --- |
| **Gene**  **IDs** | **SNP IDs** | **Allele** | **AA**  **change** | **Tolerance**  **Index** | **Prediction** | **PSIC** | **Prediction** | **DDG** | **Prediction** | **subPSEC** | **Prediction** | **References** |
| ***G6PD*** | rs1050827 | C/G | Q11H | 0.01 | Deleterious | 1.858 | Damaging | -1.52 | Decrease stability | -2.11008 | Tolerated | NA |
|  | rs78478128 | C/G | A44G | 0.00 | Tolerated | 2.178 | Damaging | -0.48 | Decrease stability | -5.29666 | Deleterious | [28] |
|  | rs76645461 | A/G | I48T | 0.10 | Deleterious | 1.36 | Benign | -2.05 | Decrease stability | -2.35477 | Tolerated | [29] |
|  | rs77362384 | C/T | D58E | 0.02 | Deleterious | 0.224 | Benign | -0.38 | Decrease stability | -2.21333 | Tolerated | NA |
|  | rs1050828 | A/G | V68M | 0.01 | Deleterious | 0.703 | Benign | -3.32 | Decrease stability | -3.99356 | Deleterious | [35] |
|  | rs11555344 | G/T | V77L | 0.12 | Tolerated | 0.196 | Benign | -0.35 | Decrease stability | -3.24632 | Deleterious | NA |
|  | rs5030870 | A/G | D113N | 0.13 | Tolerated | 0.265 | Benign | -1.58 | Decrease stability | -3.0876 | Deleterious | NA |
|  | rs1050829 | A/G | N126D | 0.50 | Tolerated | 0 | Benign | -1.14 | Decrease stability | -2.14919 | Tolerated | [35] |
|  | rs78365220 | C/T | L128P | 0.02 | Deleterious | 1.219 | Benign | 0.11 | Increase stability | -4.33811 | Deleterious | NA |
|  | rs5030872 | A/T | D181V | 0.22 | Tolerated | 0.158 | Benign | -1.20 | Decrease stability | -2.57566 | Tolerated | NA |
|  | rs74575103 | A/G | R285H | 0.00 | Deleterious | 1.03 | Benign | -1.86 | Decrease stability | -4.27988 | Deleterious | NA |
|  | rs76723693 | C/T | L323P | 0.07 | Deleterious | 1.464 | Benign | -2.07 | Decrease stability | -4.28676 | Deleterious | NA |
|  | rs5030869 | A/G | A335T | 0.00 | Deleterious | 0.564 | Benign | -0.60 | Decrease stability | -2.66901 | Tolerated | NA |
|  | rs34193178 | C/G | D350H | 0.00 | Deleterious | 0.167 | Benign | -2.00 | Decrease stability | -3.47855 | Deleterious | NA |
|  | rs72554665 | C/G/T | R459P | 0.04 | Deleterious | 1.824 | Damaging | -1.59 | Decrease stability | -3.92917 | Deleterious | NA |
|  | rs72554664 | A/G | R463H | 0.00 | Deleterious | 0.5 | Benign | -1.58 | Decrease stability | -4.20661 | Deleterious | NA |
| ***PKLR*** | rs118204087 | A/G | G37E | 0.00 | Deleterious | 1.66 | Damaging | 0.27 | Increase stability | -1.16126 | Tolerated | [30] |
|  | rs118204089 | C/G | S130Y | 0.00 | Deleterious | 0.972 | Benign | 0.33 | Increase stability | -3.27966 | Deleterious | [31] |
|  | rs118204083 | C/T | R163C | 0.00 | Deleterious | 3.591 | Damaging | -0.20 | Decrease stability | -7.83896 | Deleterious | [30] |
|  | rs74315362 | C/T | T384M | 0.00 | Deleterious | 2.871 | Damaging | 0.03 | Increase stability | -8.05846 | Deleterious | [32] |
|  | rs118204084 | A/C | Q421K | 0.01 | Deleterious | 1.46 | Benign | -0.33 | Decrease stability | -4.4669 | Deleterious | [33] |
|  | rs118204085 | A/G | R479H | 0.10 | Tolerated | 0.208 | Benign | -0.52 | Decrease stability | -3.21646 | Deleterious | [34], [35] |
|  | rs116100695 | A/G | R486W | 0.00 | Deleterious | 2.827 | Damaging | 0.35 | Increase stability | -6.66319 | Deleterious | [34], [36] |
|  | rs117089358 | A/G | R490W | 0.02 | Deleterious | 2.28 | Damaging | -0.40 | Decrease stability | -6.4158 | Deleterious | NA |
|  | rs113403872 | C/T | R510Q | 0.00 | Deleterious | 1.063 | Benign | -0.66 | Decrease stability | -3.94634 | Deleterious | [36], [37] |
| ***PKM2*** | rs11558365 | G/T | Q16H | 0.28 | Tolerated | 2.047 | Damaging | 0.01 | Increase stability | -2.78278 | Tolerated | NA |
|  | rs11558360 | A/G | E28K | 0.01 | Deleterious | 1.642 | Damaging | -0.59 | Decrease stability | -2.36591 | Tolerated | NA |
|  | rs11558375 | G/T | C31F | 0.00 | Deleterious | 2.879 | Damaging | -0.36 | Decrease stability | -3.5341 | Deleterious | NA |
|  | rs61753428 | C/T | N155S | 0.44 | Tolerated | 0.155 | Benign | 0.26 | Increase stability | -2.76397 | Tolerated | NA |
|  | rs11558351 | C/T | K186N | 0.49 | Tolerated | 0.744 | Damaging | -0.11 | Decrease stability | -3.60836 | Deleterious | NA |
|  | rs11558354 | G/T | G200C | 0.00 | Deleterious | 2.352 | Damaging | -1.57 | Decrease stability | -6.1558 | Deleterious | NA |
|  | rs17853396 | A/C | G204V | 0.00 | Deleterious | 2.013 | Damaging | -0.56 | Decrease stability | -4.87746 | Deleterious | [38] |
|  | rs11558370 | A/C | Q310P | 0.00 | Deleterious | 3.375 | Damaging | -2.2 | Decrease stability | -7.51475 | Deleterious | NA |
|  | rs2959910 | C/G | R339P | 0.00 | Deleterious | 2.06 | Damaging | -1.69 | Decrease stability | -5.79762 | Deleterious | NA |
|  | rs59430203 | G/T | S437V | 0.00 | Deleterious | 2.013 | Damaging | -1.05 | Decrease stability | -2.2664 | Tolerated | NA |
|  | rs11558358 | G/T | V490L | 0.75 | Tolerated | 0.26 | Benign | -0.66 | Decrease stability | -3.21601 | Deleterious | NA |

**(DDG < 0); Increase stability (DDG > 0); PANTHER subPSEC score: Deleterious (> -3); Tolerated (< -3).**
