# Supplementary material for: Path to Facilitate the Prediction of Functional Amino Acid Substitutions in Red Blood Cell Disorders – A Computational Approach
Source: PLoS One. 2011 Sep 13;6(9):e24607. doi: 10.1371/journal.pone.0024607 (PMC3172254; doi:10.1371/journal.pone.0024607)
Supplement: Table S2 — Functional significance of SNPs found in untranslated region of G6PD and isoforms of PK genes by UTRscan. (DOC) [file pone.0024607.s002.doc]

**Table S2. Functional significance of SNPs found in untranslated region of *G6PD* and isoforms of *PK*** genes by UTRScan.

| **Gene IDs** | **SNP id** | **Nucleotide change** | **UTR position** | **Functional element change** |
| --- | --- | --- | --- | --- |
| ***G6PD*** | rs111776132 | C/T | 3 | Musashi binding element |
|  | rs12688181 | G/T | 3 | Internal ribosome entry site |
|  | rs11550366 | C/G | 3 | Internal ribosome entry site, SXL binding site |
|  | rs11550365 | C/T | 3 | Terminal Oligopyrimidine Tract |
|  | rs3179869 | A/G | 3 | Terminal Oligopyrimidine Tract |
|  | rs3174486 | C/T | 3 | Internal ribosome entry site |
|  | rs1050757 | A/G | 3 | Polyadenylation Signal, Terminal Oligopyrimidine Tract |
|  | rs1050047 | A/G | 3 | Internal ribosome entry site, K-BOX |
|  | rs1048969 | A/G | 3 | Polyadenylation Signal |
|  | rs111827785 | C/T | 5 | Internal ribosome entry site |
|  | rs78500702 | C/G | 5 | Internal ribosome entry site |
|  | rs73641104 | C/T | 5 | Musashi binding element |
|  | rs56262786 | C/G | 5 | Musashi binding element, K-BOX |
|  | rs5986992 | A/C | 5 | Musashi binding element, K-BOX |
| ***PKLR*** | rs58009547 | C/Y | 5 | K-BOX, Musashi binding element |
|  | rs41264939 | A/C | 5 | Selenocysteine Insertion Sequence-type1 (SECIS1), Musashi binding element |
|  | rs8177998 | A/G | 5 | Polyadenylation Signal |
|  | rs8177997 | G/T | 5 | Musashi binding element |
|  | rs8177996 | C/T | 5 | Musashi binding element |
|  | rs1052177 | C/T | 5 | Internal ribosome entry site |
|  | rs8847 | A/G | 5 | UNR binding site, Musashi binding element |
| ***PKM2*** | rs11558374 | A/C | 3 | Internal ribosome entry site, Musashi binding element |
|  | rs8192432 | C/G | 3 | Internal ribosome entry site |
