# Supplementary material for: Path to Facilitate the Prediction of Functional Amino Acid Substitutions in Red Blood Cell Disorders – A Computational Approach
Source: PLoS One. 2011 Sep 13;6(9):e24607. doi: 10.1371/journal.pone.0024607 (PMC3172254; doi:10.1371/journal.pone.0024607)
Supplement: Table S3 — List of nsSNPs and UTR SNPs found to be functionally significant by FASTSNP. (DOC) [file pone.0024607.s003.doc]

**Table S3.** List of nsSNPs and UTR SNPs found to be functionally significant by FASTSNP.

| **Gene IDs** | **SNP IDs** | **Level of risk** | **region** | **Possible functional effect** |
| --- | --- | --- | --- | --- |
| ***G6PD*** | rs5030868 | Medium to high (3-4) | Coding | Splicing regulation |
|  | **rs1050828** | Medium to high (3-4) | Coding | Splicing regulation |
|  | **rs72554664** | Low medium (2-3) | Coding | Splicing regulation |
|  | **rs72554665** | Low medium (2-3) | Coding | Splicing regulation |
|  | **rs34193178** | Low medium (2-3) | Coding | Splicing regulation |
|  | **rs5030869** | Low medium (2-3) | Coding | Splicing regulation |
|  | **rs5030872** | Low medium (2-3) | Coding | Splicing regulation |
|  | **rs1050829** | Low medium (2-3) | Coding | Splicing regulation |
|  | **rs5030870** | Low medium (2-3) | Coding | Splicing regulation |
|  | **rs11555344** | Low medium (2-3) | Coding | Splicing regulation |
|  | **rs1050827** | Low medium (2-3) | Coding | Splicing regulation |
| ***PKLR*** | rs61755431 | Low medium (2-3) | Coding | Splicing regulation |
|  | rs8177988 | Low medium (2-3) | Coding | Splicing regulation |
| ***PKM2*** | rs11558352 | Very High (5-5) | Coding | Nonsense |
|  | **rs59430203** | Medium –High (3-4) | Coding | Splicing site |
|  | **rs2959910** | Medium –High (3-4) | Coding | Splicing site |
|  | **rs11558370** | Medium –High (3-4) | Coding | Splicing site |
|  | **rs17853396** | Medium –High (3-4) | Coding | Splicing site |
|  | **rs11558354** | Medium –High (3-4) | Coding | Splicing site |
|  | **rs11558375** | Medium –High (3-4) | Coding | Splicing site |
|  | **rs11558360** | Medium –High (3-4) | Coding | Splicing site |
|  | **rs11558365** | Medium –High (3-4) | Coding | Splicing site |
|  | rs11855870 | Low medium (2-3) | Coding | Splicing regulation |
|  | **rs11558358** | Low medium (2-3) | Coding | Missense (conservative) |
|  | **rs11558351** | Low medium (2-3) | Coding | Splicing regulation |

**rs IDs which are highlighted in bold were predicted to be functionally significant by SIFT/PolyPhen/PANTHER/I-Mutant 2.0**
